# A U-shaped dose–response of carbohydrate–protein supplementation on rowing performance

**DOI:** 10.3389/fnut.2025.1651457

**Published:** 2025-10-24

**Authors:** Xiangyu Wang, Hao Wu

**Affiliations:** ^1^Department of Physical Education, Capital Normal University, Beijing, China; ^2^School of Kinesiology and Health, Capital University of Physical Education and Sports, Beijing, China

**Keywords:** dose–response relationship, optimal dose, carbohydrate–protein supplement, endurance performance, rowing

## Abstract

**Background and aim:**

Co-ingestion of carbohydrate and protein supplement (CHO–PRO) is a common strategy to enhance endurance performance. However, the optimal dose–response relationship has not been established, which limits evidence-based nutritional guidance for individuals. This study aimed to characterize the performance dose–response curve of a 4:1 CHO–PRO during prolonged rowing.

**Methods:**

In a randomized, double-blind, parallel-group trial, 171 physically active male university students (age: 23 ± 2 years) from non-sports majors each completed a single experimental session. Each session involved a rowing protocol consisting of two 30-min bouts. During the exercise, participants consumed one of eight distinct doses of a 4:1 CHO–PRO in aliquots every 15 min. The CHO delivery rates ranged from 0.5 to 1.2 g/kg/h. Total rowing distance served as the primary performance outcome and was analyzed using a one-way ANCOVA with baseline countermovement jump as a covariate.

**Result:**

A significant quadratic (U-shaped) dose–response relationship was found for rowing performance. The lowest dose CHO–PRO (0.5 g/kg/h CHO) resulted in significantly greater rowing distance compared to several higher doses (0.9–1.2 g/kg/h). No significant main effect of supplement dose was observed for heart rate, blood lactate, blood glucose, or rating of perceived exertion during exercise. Post-exercise recovery markers also did not differ significantly between the groups.

**Conclusion:**

For prolonged rowing, a lower dose of the CHO–PRO was more effective than higher doses, revealing a non-linear performance response. This non-linear response was characterized by significant performance decrements at several higher intake levels. These findings underscore the importance of dose optimization. Exceeding a certain intake threshold may impair endurance performance.

## 1 Introduction

Sustaining high-power output during prolonged exercise represents a fundamental challenge in athletic performance. The depletion of endogenous carbohydrate (CHO) stores is a primary factor limiting endurance (1–3). Consequently, exogenous CHO supplementation is a well-established strategy to mitigate fatigue and extend performance (4, 5). The co-ingestion of protein (PRO) with CHO may offer synergistic benefits for substrate utilization (6, 7). Therefore, systematically evaluating the dose–response effects of various carbohydrate–protein supplement (CHO–PRO) quantities on endurance performance is crucial for developing optimal nutritional strategies.

The ergogenic role of exogenous CHO supplementation during prolonged exercise is well-established. Consensus guidelines recommend specific intake rates to maintain performance; for exercise lasting over 2 h, this intake is typically between 60 and 90 grams per hour (2, 4). To further enhance performance, the co-ingestion of PRO with CHO has been investigated. Several studies and systematic reviews suggest this combination can prolong time to exhaustion more effectively than CHO alone (8–10). Proposed physiological mechanisms for this advantage are multifactorial. The addition of PRO may augment the insulin response to CHO intake (11, 12). This action can enhance muscle glucose uptake and potentially spare endogenous glycogen stores. Additional evidence indicates that CHO–PRO may also attenuate markers of post-exercise muscle damage (10, 13). Within this strategy, specific CHO: PRO ratios are often emphasized. In particular, a ratio of approximately 3:1 to 4:1 has been proposed to optimize endurance performance and glycogen resynthesis (14, 15).

Despite the general consensus on nutrient ratios, the optimal absolute dosage for performance remains poorly defined. This knowledge gap stems primarily from methodological limitations in the existing literature. Most investigations employ a “point-to-point” design, comparing a single dose of a CHO–PRO against a CHO-only or placebo condition (12, 16). This approach cannot establish a clear dose–response relationship, leaving the optimal intake level undefined. Determining this relationship is critical; insufficient dosage may fail to yield maximal benefits, while excessive amounts provide no additional advantage and may increase the risk of gastrointestinal (GI) distress. The literature also contains inconsistencies regarding the benefits of CHO–PRO, with underlying mechanisms not yet clearly defined (13). Although a few studies have compared different supplement concentrations (13, 17, 18), systematic research across a wide range of doses remains scarce. Furthermore, much of the existing research has focused on endurance sports such as cycling and running (19, 20). In contrast, systematic dose–response investigations of CHO–PRO in rowing are notably absent from the literature (6). This omission is critical due to the unique physiological demands of rowing. The sport is a total-body exercise, engaging a large muscle mass across both the upper and lower limbs. This extensive muscle recruitment leads to a high metabolic rate and significant cardiovascular strain. Consequently, substrate utilization patterns and nutritional requirements in rowing may differ from those in predominantly lower-body sports. Therefore, generalizing findings from cyclists or runners to rowers is inappropriate. A rigorous, rowing-specific study is essential to systematically evaluate the dose–response effects of CHO–PRO on endurance performance.

Therefore, the primary aim of this study was to characterize the dose–response curve for a 4:1 CHO–PRO on prolonged rowing performance. To move beyond simple point-to-point comparisons, a multi-dose experimental design was employed to model this relationship across a range of intake levels. It was hypothesized that performance would exhibit a non-linear, quadratic relationship with supplement dosage. The findings are intended to provide evidence-based guidance for optimizing nutritional strategies to enhance endurance rowing performance.

## 2 Methods

### 2.1 Participants

An initial pool of 230 male university students from non-sports-related majors was assessed for eligibility. Following screening with the Physical Activity Readiness Questionnaire (PAR-Q), 195 individuals were deemed eligible to participate. A final sample of 171 participants successfully completed all experimental procedures and their data were included in the final analysis. The study employed a between-subjects design. Each participant completed only one experimental trial. Specific inclusion criteria required participants to be physically active, defined as engaging in structured exercise at least three times per week for more than 60 min per session (21). The nature of this structured exercise was recreational, not professional. Additionally, all individuals had to be familiar with the use of a rowing ergometer sufficient to maintain a stable technique throughout the protocol. Exclusion criteria included any contraindication to exercise identified by the PAR-Q (22), a history of severe organ dysfunction, or current adherence to restrictive dietary protocols. Before the study, participants were informed of the research purpose, experimental procedures, and potential risks, after which they provided written informed consent. The study was approved by the Ethics Committee of Capital University of Physical Education and Sports (No. 2022A57). The basic information of the subjects is shown in Table 1.

**Table 1:** Baseline characteristics of the participants (*N* = 171).

Characteristic	Unit	Mean ± SD
Age	years	23 ± 2
Height	cm	179.2 ± 6.9
Weight	kg	76.1 ± 9.9
Body Fat %	%	17.0 ± 5.0
CMJ	cm	37.5 ± 6.4

### 2.2 Exercise protocol

**Figure 1:**
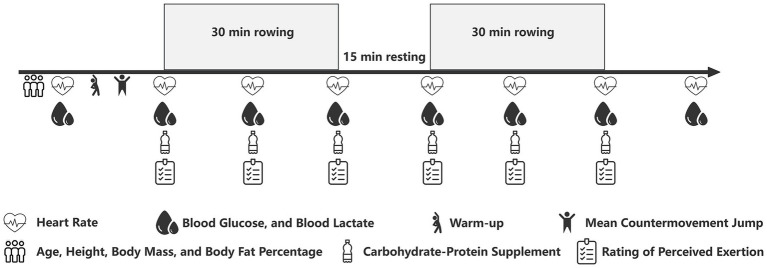
Schematic of the experimental protocol.

The performance protocol was adapted from a standardized 1-h rowing test to assess total work capacity over a prolonged duration (6, 23). The protocol consisted of two 30-min rowing bouts on a Concept II (Type D) rowing ergometer, separated by a 15-min rest period (Figure 1). This intermittent design was implemented following pilot testing with individuals representative of the study’s target population. Pilot observations revealed that a continuous 60-min high-intensity row was not feasible for this cohort. Specifically, substantial decrements in power output and a deterioration in technique were noted in the latter stages of a continuous bout. The adopted 2 × 30 min format was therefore chosen to ensure participants could maintain a high and consistent work rate throughout the total exercise duration. The ergometer was programmed with a 30-min countdown for each bout to ensure uniform exercise duration for all participants.

To standardize test conditions and elicit maximal effort, the ergometer drag factor was set to 120 for all participants. Standardized verbal encouragement was provided throughout the test. The ergometer’s display monitor was obscured from the participant’s view to prevent pacing adjustments based on distance feedback and to minimize inter-participant comparison. Participants were instructed to maintain a stroke rate between 16 and 24 strokes per minute and to find a maximal yet sustainable power output for the entirety of the test.

Pre-defined termination criteria were established to ensure participant safety and test validity. The test was to be stopped if a participant’s heart rate (HR) dropped below 40% of their HR reserve on three separate occasions (24, 25). Other criteria for termination included signs of angina, shortness of breath, wheezing, muscle cramps, light-headedness, confusion, ataxia, pallor, cyanosis, nausea, or cold and clammy skin. A test would also be concluded if the participant showed signs of extreme fatigue, requested to stop, or if there was an equipment malfunction.

### 2.3 Nutritional strategy

The CHO and PRO supplements were sourced from ALL STARS (Germany) (6). The PRO was a banana-sundae-flavored whey protein hydrolysate powder, and the CHO was an orange-flavored powdered drink mix. The CHO component consisted of corn starch (46%), glucose (40.5%), and maltose (5%). All supplements were formulated with a fixed CHO: PRO ratio of 4:1. Eight distinct supplement doses were created based on an established range of effective CHO intake (6, 26). The CHO delivery rates ranged from 0.5 to 1.2 g/kg/h. The relative and absolute dosages for each condition are presented in Table 2. For each participant’s experimental session, the assigned substance was prepared as a 900 mL beverage. This combination resulted in a pale-yellow, opaque liquid, which helped to conceal visual differences between doses. The beverage was consumed in 150 mL aliquots every 15 min during exercise, with each aliquot served in an opaque, lidded container. A randomized, double-blind, parallel-group design was employed. A computer-generated random sequence was used to assign participants to one of eight supplement conditions. Block randomization was implemented to ensure balanced group sizes throughout the study. The block size was set to eight, containing one allocation for each of the eight supplement conditions. The order of allocations within each block was randomized. The eight active supplement doses were assigned numerical codes from 1 to 8. The codes did not correspond to the incremental order of the dosages. Both participants and research staff who administered the supplements were blinded to the group assignments.

**Table 2:** Details of the eight supplementation conditions.

Condition code	CHO dose (g/kg/h)	Mean absolute CHO intake (g)	PRO dose (g/kg/h)	Mean absolute PRO intake (g)
1	0.5	50.85 ± 5.16	0.125	12.71 ± 1.29
2	0.6	55.30 ± 6.00	0.15	13.83 ± 1.50
3	0.7	66.93 ± 11.15	0.175	16.73 ± 2.79
4	0.8	78.33 ± 13.46	0.2	19.58 ± 3.36
5	0.9	83.79 ± 7.79	0.225	20.95 ± 1.95
6	1	96.20 ± 11.39	0.25	24.05 ± 2.85
7	1.1	99.72 ± 12.27	0.275	24.93 ± 3.07
8	1.2	111.67 ± 11.33	0.3	27.92 ± 2.83

### 2.4 Experimental procedures and data collection

One week before the formal trial, all participants attended a familiarization session. This session was designed for participants to practice the exercise protocol and the beverage consumption schedule using plain water. During this session, they were also instructed on the pre-trial control procedures for the 48 h preceding their experiment. Participants were required to replicate their typical dietary patterns and to avoid any strenuous or unaccustomed physical activity.

To ensure compliance, participants documented their food intake and physical activity in a standardized log. On the day of the trial, research staff reviewed each log to confirm adherence to the instructions. This review served as a screening tool to maintain baseline validity across the cohort. A trial would be rescheduled if the log revealed: (1) any session of high-intensity or prolonged exercise, or (2) extreme deviations from a normal dietary pattern, such as fasting, excessive energy intake, or high alcohol consumption. The dietary logs also confirmed that no participants were following restrictive diets, such as ketogenic or very-low-CHO protocols.

All experimental trials were conducted between May and August 2022. To facilitate the recruitment of a large sample for subsequent machine learning applications (6), testing sessions were scheduled flexibly between 08:00 and 17:00. This approach aimed to capture performance data under a range of real-world conditions. Participant scheduling was contingent upon their strict adherence to all pre-trial control procedures described previously. A maximum of three participants were tested concurrently in the laboratory.

Seven days after the familiarization session, each participant attended the laboratory to complete their single, formal experimental trial. Upon arrival at the laboratory, baseline data including age, height (Suhong, China) (27), body mass, and body fat percentage (InBody 270, Biospace, Korea) (28) were recorded. Resting HR (Polar H10, Kempele, Finland) (29), blood glucose (BG; Sinocare Anwen+, Changsha, China) (30), and blood lactate (BLa; EKF Lacte Scout 4, Barleben, Germany) (31) were also measured. Following these initial measurements, participants performed a standardized 5-min warm-up. Mean countermovement jump (CMJ) (Omegawave system, Espoo, Finland) (32) was then assessed as the average of five consecutive jumps.

During the exercise protocol, data were collected at 15-min intervals. Rating of perceived exertion (RPE), HR, BG, and BLa were measured at the following time points: immediately before exercise, and at 15, 30, 45, 60, and 75 min (upon completion of the protocol), totaling six collection points. A final measurement of HR, BG, and BLa was taken 15 min after exercise cessation.

### 2.5 Statistical analysis

All statistical analyses were performed using Python with core scientific libraries. Data are presented as mean ± standard deviation (SD). Assumptions of normality and homogeneity of variances were assessed using the Shapiro–Wilk and Levene’s tests. A one-way ANCOVA, with baseline CMJ as a covariate, was used to analyze total rowing distance. A polynomial contrast then tested for a quadratic dose–response relationship.

Physiological responses (HR, BLa, BG, RPE) during exercise were analyzed in two separate blocks using linear mixed-effects models (LMMs). For the first exercise bout, the model assessed responses at 15 and 30 min. The corresponding 0 min baseline value was included as a covariate. For the second bout, the model assessed responses at 60 and 75 min, with the 45 min value serving as a covariate. Both LMMs treated supplement dose, time, and the dose-by-time interaction as fixed effects. A random intercept for each participant was included in the model.

Changes in markers during the recovery period (30–45 min) were assessed using one-way ANOVA. Post-exercise data, collected at a single time point, were also compared between groups using one-way ANOVA. Effect sizes for ANOVA were calculated using eta-squared (*η*^2^). Significant main effects were explored using Tukey’s HSD post-hoc tests. The Benjamini–Hochberg procedure was applied to control the false discovery rate for multiple comparisons. Statistical significance was set at *p* < 0.05.

A sensitivity power analysis (G*Power, version 3.1.9.7) was performed. For a one-way ANCOVA with eight groups and one covariate, the sample size (*N* = 171) provided 80% power to detect a minimum effect size of *f* = 0.296 at an alpha level of 0.05. The primary analysis revealed an observed effect of *ω*^2^ = 0.061 (*f* ≈ 0.255) for the main intervention. The observed effect was smaller than the minimum detectable effect size. Therefore, the study was underpowered for the magnitude of the effect found.

## 3 Results

Prior to all inferential statistical analyses, the relevant dependent variables were confirmed to meet the assumptions of normality (Shapiro–Wilk test, *p* > 0.05) and homogeneity of variances (Levene’s test, *p* > 0.05). Additionally, there were no significant differences in the baseline indicators among the groups before the exercise.

### 3.1 Effect of supplementation on rowing performance

**Figure 2:**
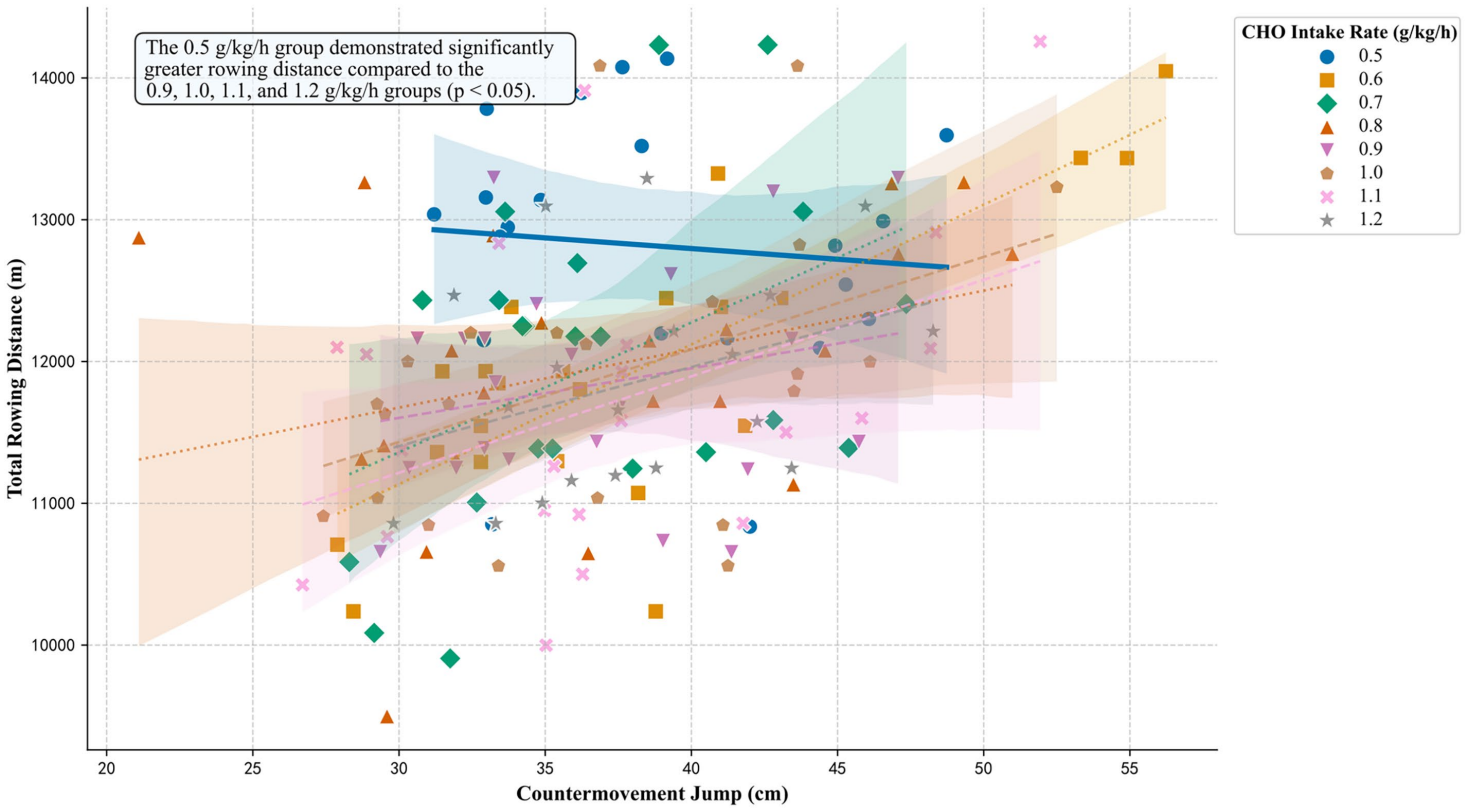
Rowing performance as a function of CMJ and supplement dose. The plot visualizes the ANCOVA model. Each point represents an individual participant. Data are grouped by the CHO intake rate. Each group is distinguished by a unique symbol and regression line style to ensure clarity in both color and grayscale formats. The regression line for the 0.5 g/kg/h dose is highlighted with a thicker line, reflecting its significantly greater performance. An in-plot annotation explicitly states the primary statistical finding. Shaded areas represent the 95% confidence intervals for the regression fits.

An ANCOVA was conducted to determine the effect of different CHO intake rates on rowing distance, with CMJ height as a covariate. The analysis revealed a significant main effect for the covariate (CMJ) on rowing distance (*F* (1, 162) = 29.86, *p* < 0.001, *ηp*^2^ = 0.16, 95% CI [0.01, 1.00]). A significant main effect was also found for CHO intake rate (*F* (7, 162) = 2.54, *p* = 0.016, *ηp*^2^ = 0.10, 95% CI [0.08, 1.00]). The wide confidence interval for this effect size indicates considerable uncertainty in its true magnitude. The relationship between rowing distance, CMJ, and CHO intake rate is visualized in Figure 2. The regression line for the 0.5 g/kg/h CHO intake rate group, while positioned highest overall, exhibits a slight negative slope.

Post-hoc comparisons using Tukey’s HSD test were performed to identify specific group differences. The 0.5 g/kg/h group demonstrated a significantly greater rowing distance compared to four of the higher-dose groups. Significant differences were found against the 0.9 g/kg/h (*p* = 0.025; Hedges’ *g* = 1.12, 95% CI [0.47, 1.76]), 1.0 g/kg/h (*p* = 0.045; Hedges’ *g* = 0.94, 95% CI [0.30, 1.57]), 1.1 g/kg/h (*p* = 0.007; Hedges’ *g* = 1.07, 95% CI [0.42, 1.71]), and 1.2 g/kg/h (*p* = 0.045; Hedges’ *g* = 1.10, 95% CI [0.43, 1.76]) conditions. No other significant differences were detected between any other pairs of groups (all *p* > 0.05).

### 3.2 Dose–response trend analysis

**Figure 3:**
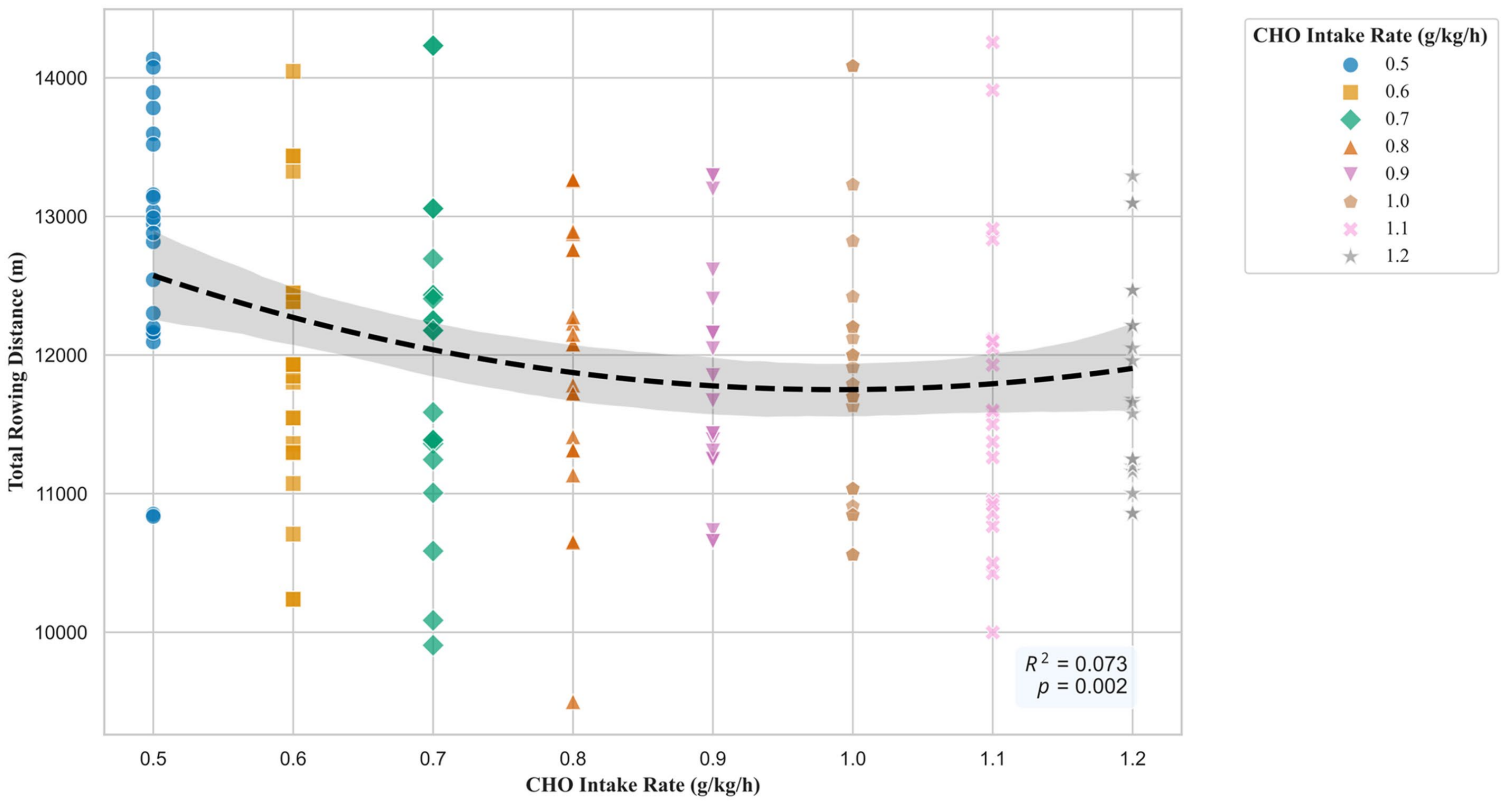
Quadratic dose–response relationship between CHO supplementation and rowing performance. Individual data points are shown. Each of the eight dose groups is represented by a unique symbol and color to visualize the group relationships within the overall trend. The dashed black line is the best-fit quadratic regression model, and the shaded area indicates the 95% confidence interval. The model’s coefficient of determination (*R*^2^) and overall *p*-value are annotated on the plot.

A polynomial regression analysis was performed to determine the shape of the dose–response relationship between CHO intake rate and rowing distance. The overall quadratic model was statistically significant (*F* (2, 168) = 6.60, *p* = 0.002) and explained 7.3% of the variance in rowing distance (*R*^2^ = 0.073). The results indicated a significant linear term (*p* = 0.016) and a significant quadratic term (*p* = 0.038). The resulting dose–response curve is presented in Figure 3. The curve demonstrates a U-shaped relationship, with performance decreasing from the 0.5 g/kg/h dose to a nadir at approximately 1.0 g/kg/h, before increasing at higher dosages.

### 3.3 Physiological and perceptual responses to supplementation

#### 3.3.1 Glycemic control

The analysis of BG was conducted separately for the two 30-min exercise bouts and the intervening recovery period.

**Figure 4:**
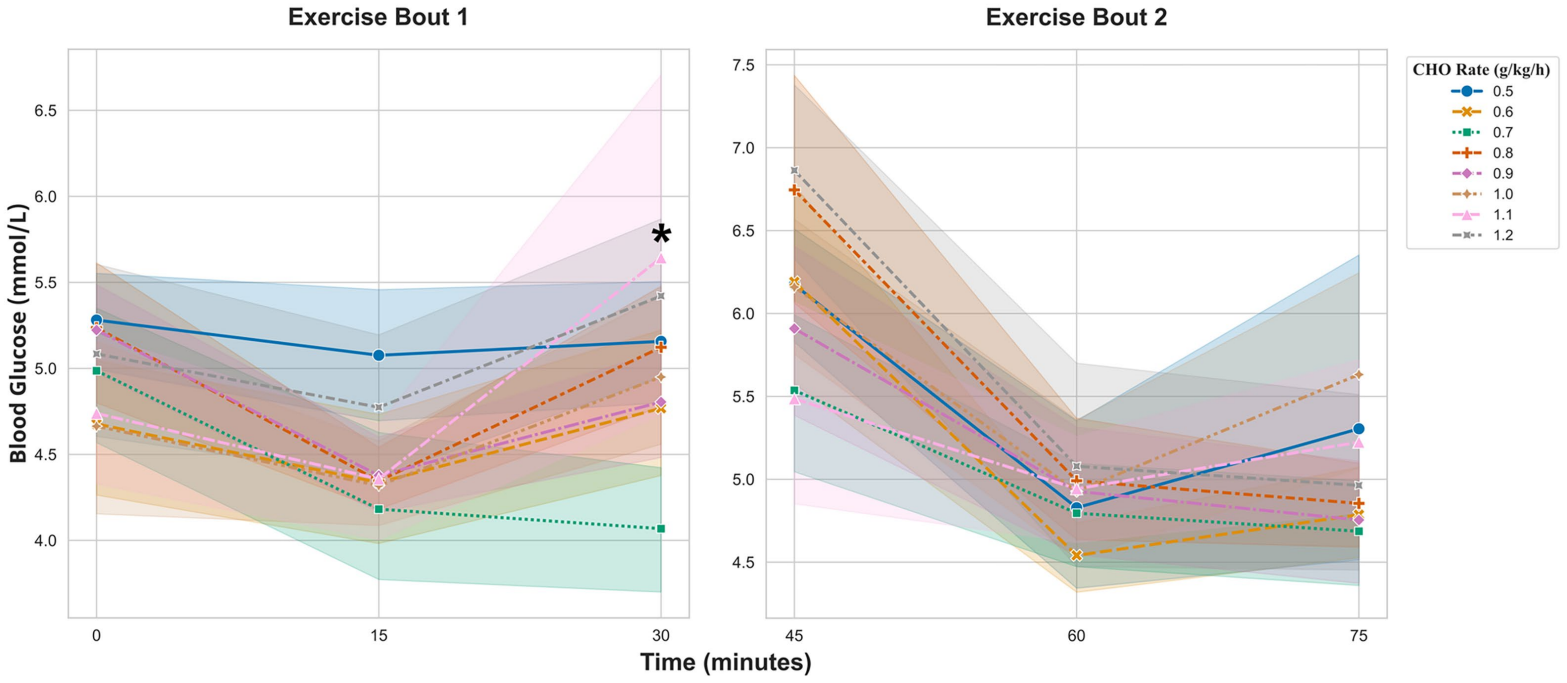
BG responses during the first and second 30-min exercise bouts. Values are presented as estimated marginal means ±95% confidence intervals, adjusted for the corresponding baseline value of each bout. *Indicates a significant dose-by-time interaction, representing a different response trajectory compared to the 0.5 g/kg/h reference group (*p* < 0.05).

During the first exercise bout (0–30 min), a significant dose-by-time interaction was detected (Figure 4 Exercise Bout 1). The glycemic response in the 1.1 g/kg/h group diverged significantly from the 0.5 g/kg/h reference group. This was evident in a significant interaction at 30 min (coefficient = 1.21 mmol/L, 95% CI [0.48, 1.93], *p* = 0.001). No other significant interactions were observed.

**Figure 5:**
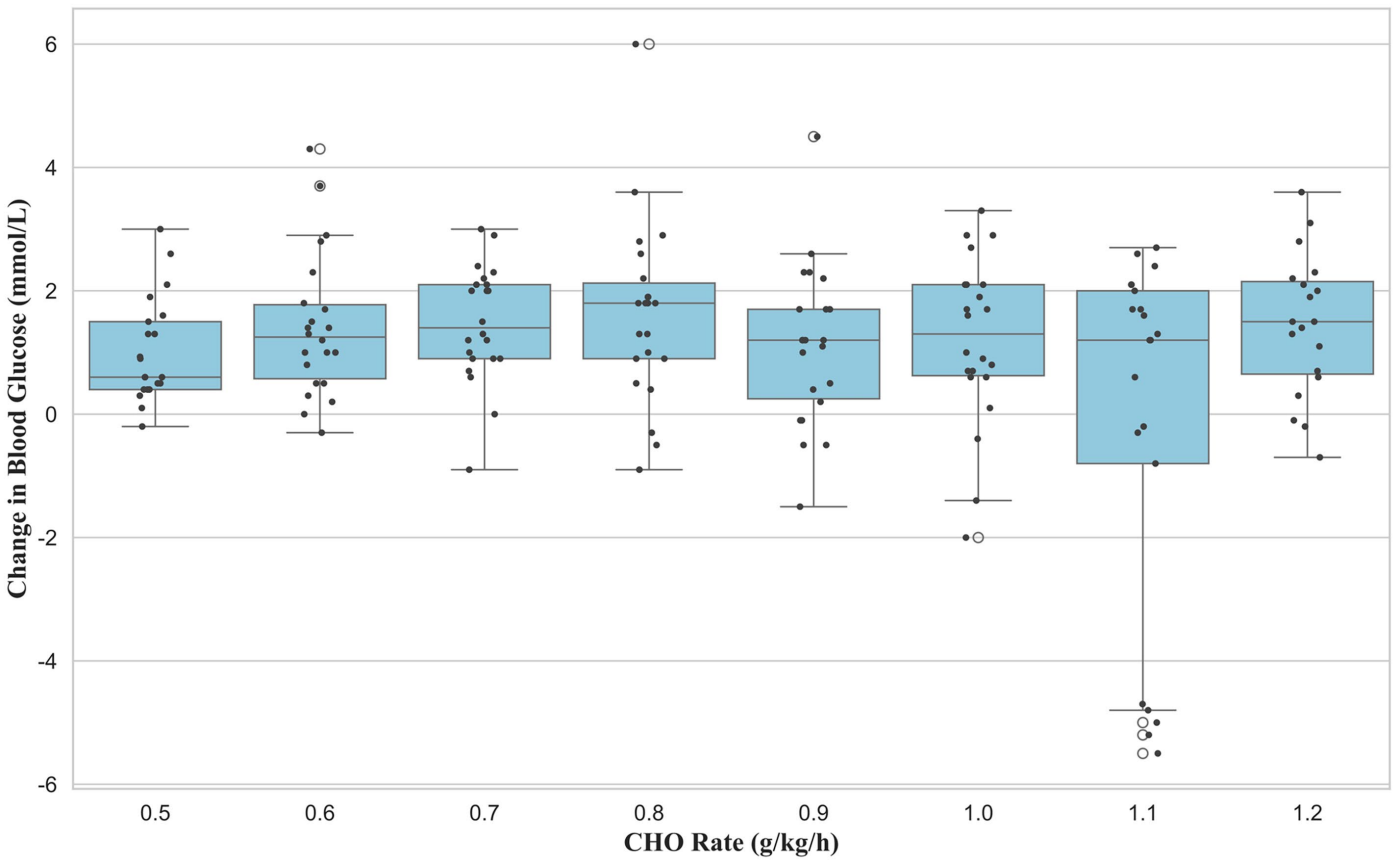
Recovery period (T45-T30): delta of BG (mmol/L).

Analysis of the change in BG during the 15-min recovery period (30–45 min) revealed a significant main effect of dose (*F* (7, 163) = 2.87, *p* = 0.008, *ηp*^2^ = 0.11, 95% CI [0.003, 0.015]). However, post-hoc tests with Benjamini–Hochberg correction for multiple comparisons found no significant pairwise differences between the groups (Figure 5).

#### 3.3.2 BLa dynamics

BLa responses during the exercise and recovery periods are presented in Table 3. During the first exercise bout (0–30 min), a significant main effect of supplementation was observed. Several doses (0.6, 0.7, 0.9, 1.1, and 1.2 g/kg/h) attenuated the overall BLa response compared to the 0.5 g/kg/h reference group. The pattern of BLa accumulation also differed at specific time points. Significant dose-by-time interactions were identified for the 0.8 g/kg/h (coefficient = 2.10 mmol/L, 95% CI [0.38, 3.82], *p* = 0.017) and 1.1 g/kg/h (coefficient = 2.79 mmol/L, 95% CI [1.05, 4.3], *p* = 0.002) groups, which exhibited a greater relative increase in BLa at 30 min. During the second exercise bout (45–75 min), a significant dose-by-time interaction was found for the 0.8 g/kg/h dose (coefficient = 2.72 mmol/L, 95% CI [0.76, 3.78], *p* = 0.003). This was characterized by a greater BLa accumulation at 75 min relative to the reference group. No other significant effects were detected in this period. Analysis of the change in BLa during the 15-min recovery period showed no significant effect of supplement dose (*F* (7, 163) = 2.02, *p* = 0.055, *ηp*^2^ = 0.08, 95% CI [0.00, 0.01]).

**Table 3:** Physiological and perceptual responses by supplement dose and time.

Variable	CHO_Rate	0 min	15 min	30 min	45 min	60 min	75 min
BLa	0.5	2.68 ± 1.56	7.61 ± 3.09	7.84 ± 3.33	5.47 ± 2.17	5.42 ± 2.55	5.63 ± 1.92
	0.6^†^	2.37 ± 1.14	5.27 ± 2.38	7.06 ± 3.11	3.91 ± 2.02	4.21 ± 2.68	4.3 ± 2.03
	0.7^†^	2.08 ± 0.56	5.42 ± 2.13	7.23 ± 3.16	5.2 ± 2.44	4.42 ± 2.13	5.16 ± 1.78
	0.8^†^	2.38 ± 1.29	6.09 ± 2.37	8.41 ± 3.82^*^	4.27 ± 2.05	4.33 ± 1.47	6.81 ± 4.64^*^
	0.9^†^	2.31 ± 1.02	5.74 ± 2.11	6.66 ± 2.39	4.05 ± 1.52	3.97 ± 1.51	4.6 ± 1.58
	1	2.19 ± 1.84	5.7 ± 2.12	6.62 ± 2.53	4.2 ± 2.51	4.55 ± 2.89	5.48 ± 3.34
	1.1^†^	2.98 ± 1.38	5.62 ± 2.48	8.64 ± 4.07	4.84 ± 2.27	5.19 ± 2.23	5.65 ± 2.18
	1.2	2.66 ± 1.05	5.54 ± 2.11	6.17 ± 2.83	3.91 ± 2.13	3.79 ± 2.25	4.72 ± 2.6
HR	0.5	84.43 ± 11.08	170.86 ± 13.28	180.19 ± 16.33	103.81 ± 18.89	161.48 ± 18.76	172.76 ± 16.66
	0.6^†^	83.64 ± 8.51	156.77 ± 13.12	166.09 ± 19.41	96.45 ± 14.13	154.32 ± 15.24	165.5 ± 21.63
	0.7^†^	80.59 ± 10.43	158.86 ± 19.47	173.59 ± 18.15	95.09 ± 11.78	156.0 ± 20.41	168.82 ± 20.18
	0.8	85.55 ± 14.27	167.23 ± 15.27	181.73 ± 12.72	95.82 ± 14.96	162.09 ± 12.58	175.18 ± 12.04
	0.9^†^	80.68 ± 11.01	158.95 ± 16.57	170.23 ± 18.14	98.5 ± 10.46	153.27 ± 16.65	165.68 ± 17.82
	1^†^	87.86 ± 8.72	160.0 ± 14.09	169.55 ± 17.4	102.36 ± 9.54	160.32 ± 14.95	165.05 ± 16.67
	1.1^†^	87.33 ± 14.97	158.24 ± 18.19	170.62 ± 19.77	101.05 ± 10.03	156.95 ± 17.81	168.1 ± 18.52
	1.2^†^	84.32 ± 10.31	160.0 ± 18.35	171.68 ± 17.95	99.32 ± 11.1	162.26 ± 17.85	176.16 ± 14.66
RPE	0.5	9.43 ± 2.04	14.38 ± 2.6	16.33 ± 2.35	11.67 ± 2.03	15.0 ± 1.9	16.76 ± 1.73
	0.6	9.95 ± 1.68	13.73 ± 1.93	16.09 ± 2.14	13.0 ± 2.31	15.36 ± 1.59	17.09 ± 1.02
	0.7^†^	9.09 ± 2.35	12.55 ± 1.79	16.68 ± 1.84^*^	12.27 ± 1.49	15.68 ± 1.64	17.55 ± 1.79
	0.8	9.68 ± 1.73	14.18 ± 2.04	16.73 ± 1.67	12.55 ± 1.87	15.36 ± 1.73	17.14 ± 1.46
	0.9	9.41 ± 1.79	14.64 ± 1.87	17.05 ± 1.99	11.55 ± 1.18	14.77 ± 2.05	16.73 ± 1.91
	1	9.32 ± 1.67	13.86 ± 2.87	15.77 ± 2.49	11.95 ± 2.95	15.14 ± 2.73	16.41 ± 2.52
	1.1	10.0 ± 1.79	14.43 ± 1.86	16.1 ± 1.76	11.62 ± 2.16	14.48 ± 2.09	17.05 ± 1.43
	1.2	9.11 ± 1.91	13.84 ± 2.5	16.58 ± 1.8	11.53 ± 2.04	15.37 ± 2.11	17.16 ± 1.89

Data are presented as mean ± standard deviation. ^†^Indicates a significant main effect for the supplement dose compared to the 0.5 g/kg/h reference group during the first exercise bout (*p* < 0.05). ^*^Indicates a significant dose-by-time interaction relative to the 0.5 g/kg/h reference group (*p* < 0.05).

#### 3.3.3 Cardiovascular strain

HR data from the exercise and recovery periods are summarized in Table 3. During the first exercise bout (0–30 min), a significant main effect of supplementation was found. Several doses (0.6, 0.7, 0.9, 1.0, 1.1, and 1.2 g/kg/h) attenuated the overall cardiovascular strain, reflected by a lower mean HR compared to the 0.5 g/kg/h reference group. No significant dose-by-time interactions were detected, indicating that the HR response trajectories were parallel across all groups. During the second exercise bout (45–75 min), no significant main or interaction effects were observed for HR. Analysis of the change in HR during the 15-min recovery period revealed a significant main effect of dose (*F* (7, 163) = 2.91, *p* = 0.007, *ηp*^2^ = 0.11, 95% CI [0.003, 0.015]). However, similar to the glycemic response, post-hoc tests with Benjamini–Hochberg correction found no significant pairwise differences between the groups.

#### 3.3.4 Perceptual response

The RPE for each condition is reported in Table 3. During the first exercise bout (0–30 min), the 0.7 g/kg/h dose had a significant main effect, corresponding to a lower overall RPE compared to the 0.5 g/kg/h reference group. A significant dose-by-time interaction was also identified for this dose (*p* < 0.001), characterized by a greater relative increase in RPE at 30 min. No significant main or interaction effects for RPE were found during the second exercise bout (45–75 min). Furthermore, the analysis of the change in RPE during the 15-min recovery period revealed no significant effect of supplement dose (*F* (7, 163) = 1.99, *p* = 0.060, *ηp*^2^ = 0.08, 95% CI [0.00, 0.01]).

### 3.4 Rate of fatigue accumulation

**Figure 6:**
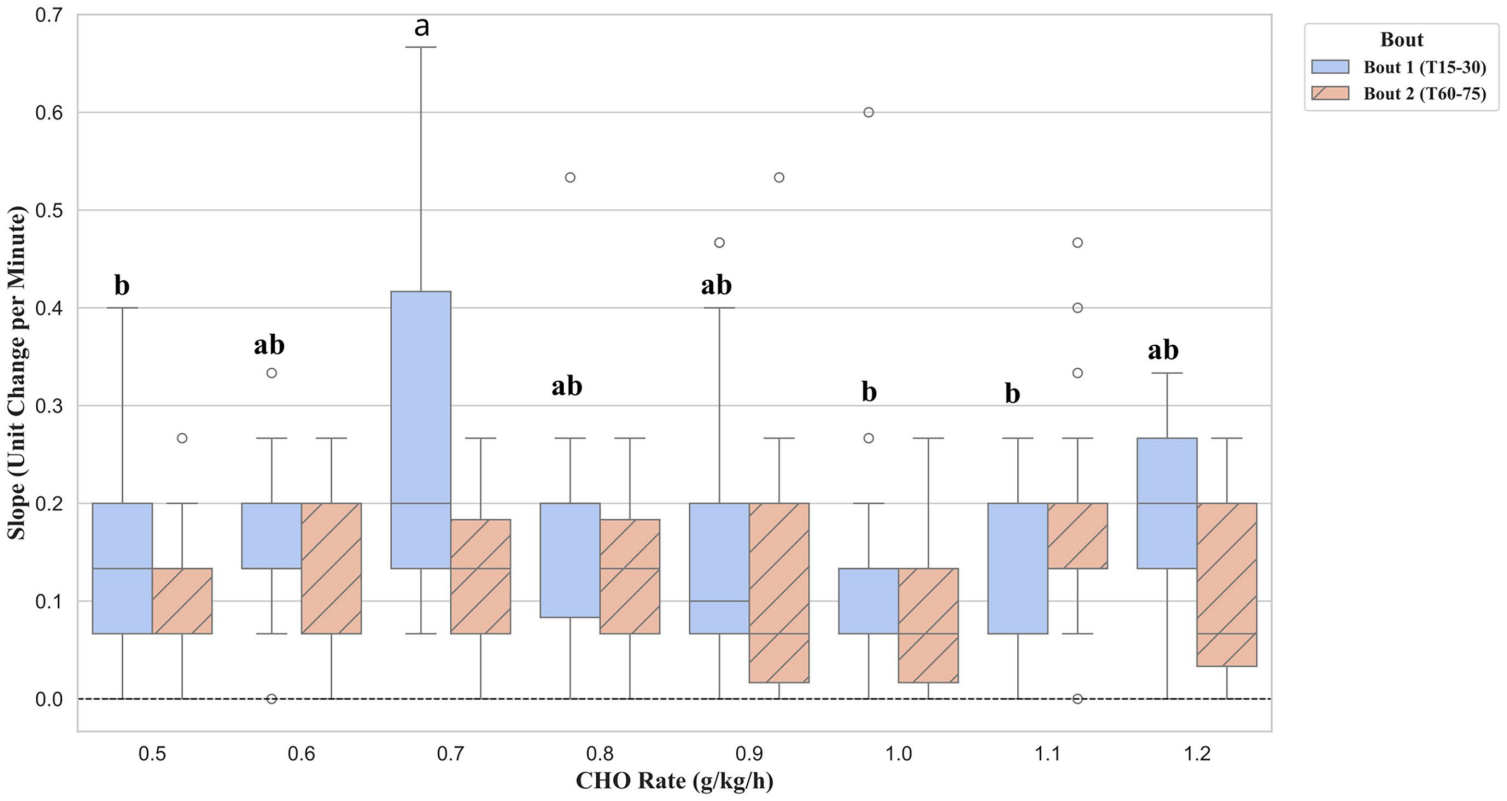
Rate of change in RPE during the first (Bout 1) and second (Bout 2) exercise bouts. Data are presented as boxplots, where the line indicates the median and the box represents the interquartile range. The rate of change was calculated as the linear slope of RPE values for each participant between 15 and 30 min (Bout 1) and between 60 and 75 min (Bout 2). Groups not sharing a common letter are significantly different within Bout 1 (*p* < 0.05), adjusted for false discovery rate.

To explore the dynamics of fatigue, the rate of change (slope) for physiological and perceptual markers was compared between the first and second exercise bouts. No significant differences between supplement groups were found for the rate of change in HR, BLa, or BG during either exercise bout. In contrast, a significant effect of supplementation was observed for the rate of RPE increase, but only during the first exercise bout (*F* (7, 163) = 3.69, *p* = 0.001, *ηp*^2^ = 0.14, 95% CI [0.008, 0.020]), as shown in Figure 6. Post-hoc analysis revealed that the 0.7 g/kg/h group exhibited a significantly faster rate of RPE accumulation compared to five other conditions. These included the 0.5 g/kg/h (*p* = 0.004, Cohen’s *d* = 0.94), 0.6 g/kg/h (*p* = 0.038, Cohen’s *d* = 0.80), 0.9 g/kg/h (*p* = 0.048, Cohen’s *d* = 0.67), 1.0 g/kg/h (*p* = 0.003, Cohen’s *d* = 0.90), and 1.1 g/kg/h (*p* < 0.001, Cohen’s *d* = 1.07).

### 3.5 Post-exercise recovery markers

Post-exercise recovery markers at 15 min are presented in Table 4. One-way ANOVAs were conducted to compare these markers across the eight CHO intake rate groups. No statistically significant differences were found for HR, BG, or BLa. The corresponding effect sizes for these null findings were small. Specific results for HR were *F* (7, 163) = 1.40, *p* = 0.210 (*ηp*^2^ = 0.06, 95% CI [0.000, 0.007]). For BG, results were *F* (7, 163) = 1.49, *p* = 0.175 (*ηp*^2^ = 0.06, 95% CI [0.000, 0.007]). For BLa, results were *F* (7, 163) = 1.30, *p* = 0.256 (*ηp*^2^ = 0.05, 95% CI [0.000, 0.006]).

**Table 4:** Physiological markers 15 min post-exercise.

CHO_Rate (g/kg/h)	HR_Post15 (bpm)	BG_Post15 (mmol/L)	BLa_Post15 (mmol/L)
0.5	88.48 ± 13.39	6.31 ± 0.89	3.45 ± 1.29
0.6	83.86 ± 10.99	6.21 ± 0.56	2.78 ± 1.07
0.7	88.27 ± 9.5	6.71 ± 0.91	2.57 ± 0.87
0.8	87.45 ± 12.81	6.81 ± 0.59	3.36 ± 1.47
0.9	90.55 ± 10.05	6.62 ± 0.92	2.78 ± 1.06
1	92.36 ± 10.04	6.69 ± 0.86	3.06 ± 2.27
1.1	88.62 ± 10.34	6.69 ± 0.94	3.66 ± 1.77
1.2	84.11 ± 12.84	6.93 ± 1.47	3.14 ± 1.97

## 4 Discussion

A U-shaped dose–response relationship was identified between CHO–PRO intake and rowing performance. The lowest dose conferred a significant performance advantage over several higher doses. This finding challenges the paradigm that progressively higher substrate availability uniformly enhances endurance. A dissociation between systemic physiological responses and whole-body performance was evident. Higher supplement doses attenuated HR response and BLa accumulation, particularly during the initial exercise phase. These physiological effects, however, did not translate into improved performance outcomes. The effects were also not sustained throughout the protocol and did not alter acute recovery markers.

This result challenges the conventional assumption that progressively increasing CHO intake up to recommended ceilings consistently enhances endurance performance. While some research supports a dose–response benefit of supplementation, a body of evidence indicates that high-dose strategies do not always translate to superior outcomes (13, 33). Several studies have reported enhanced next-day or same-day subsequent performance when a lower-CHO, PRO-inclusive supplement was used compared to an isoenergetic high-CHO alternative (3, 34, 35). The current findings align with this latter view. For prolonged, high-intensity rowing, an optimal dosing strategy may exist at a lower threshold than traditionally prescribed.

A primary mechanism that may explain the performance decrement at higher doses is an increased GI burden. Higher concentrations of CHO–PRO can increase beverage osmolality, potentially delaying gastric emptying and leading to GI distress (3, 36). Although GI symptoms were not systematically measured in this study, research by Russo et al. (37) and Rauch et al. (38) provides direct evidence that higher nutrient loads during exercise can induce greater gut discomfort and CHO malabsorption. For example, increasing CHO intake from 76 to 90 g/h resulted in greater GI symptom severity (38). While Costa et al. (39) reported that a 1.2 g/kg/h CHO + PRO beverage did not cause significant GI symptoms compared to water, this may indicate an individual tolerance threshold. Therefore, it is hypothesized that even sub-clinical GI stress in the higher-dose groups may have negatively impacted performance through vagal-afferent signaling, thereby limiting central drive and work output (40).

A complementary explanation may relate to the metabolic response to high substrate loads. High oral CHO intake can lead to pronounced insulin secretion, which alters substrate availability and utilization (41, 42). A lower CHO dose may prevent potential metabolic disruptions associated with very high intake rates. Such disruptions can include large fluctuations in BG and insulin, which may compromise metabolic efficiency. The findings of Berardi et al. (43) are consistent with this concept. In their work, a supplement with a lower CHO content resulted in superior performance compared to a higher-CHO alternative. The lower 0.5 g/kg/h dose may have provided sufficient substrate without causing the metabolic disturbances linked to higher doses. Therefore, a potential, albeit unmeasured, explanation for the performance decrement at higher intakes is a reduced metabolic efficiency over the trial duration. In conclusion, the observed U-shaped performance curve is likely multifactorial, representing a trade-off between energy delivery, GI tolerance, and metabolic efficiency.

A key finding from this study was the dissociation between transient physiological responses and final performance outcomes. Although several higher supplement doses (CHO intakes of 0.6–1.2 g/kg/h) attenuated HR and BLa at specific time points, this did not translate to improved rowing distance. This observation is critical, as it questions the reliability of using isolated physiological markers as direct surrogates for performance in prolonged, high-intensity exercise (44, 45). While markers like lactate threshold and HR are commonly used to prescribe and monitor training (46, 47), the current data demonstrate that momentary improvements in these variables do not guarantee a superior overall performance.

The divergence between performance and peripheral physiology may be explained by the interplay between central and peripheral fatigue. It is plausible that while higher doses provided some peripheral benefit (e.g., an attenuated HR response or altered muscle metabolism), they may have concurrently exacerbated central fatigue. As previously discussed, the increased GI load associated with higher-dose supplements could act as a potent stressor, signaling the central nervous system to downregulate motor output to maintain homeostasis (48, 49). This aligns with the principles of the Central Governor Model, which posits that the brain regulates exercise intensity to prevent catastrophic physiological failure (50, 51). Such a centrally-mediated regulatory action would preemptively limit work output, thus negating any potential peripheral advantages.

Furthermore, the role of perceived exertion is paramount in interpreting these findings. While a main effect for RPE was isolated to a single dose group during the first exercise bout, the supplementation strategy did not induce a widespread, systematic change in RPE across the cohort. RPE is considered a powerful, integrative psychophysiological construct that dictates exercise intensity and performance (52). The data suggest that the momentary physiological advantages offered by higher doses were insufficient to alter the athletes’ overall perception of effort. Since RPE is a key determinant of self-selected pacing and endurance capacity (52), the absence of a reduction in perceived effort across the entire trial explains why performance did not improve. Ultimately, this dissociation underscores that performance is an integrated outcome, and a holistic approach that considers central, perceptual, and peripheral factors is necessary to understand the true impact of any nutritional intervention.

The large sample size (*N* = 171), which is uncommon in exercise nutrition research, provides a robust basis for the findings. The study employed a randomized, double-blind, multi-dose, parallel-group design to minimize bias and characterize the dose–response curve in detail. Furthermore, the use of ANCOVA effectively controlled for baseline differences in physical power, isolating the effects of the nutritional intervention. Finally, the investigation of eight distinct supplement dosages allowed for a high-resolution analysis of the dose–response relationship, moving beyond simple comparisons of few conditions. Despite these strengths, several limitations must be acknowledged. The findings are specific to recreationally active, male university students and may not be generalizable to elite, female, or older athlete populations. A further statistical limitation is that the study was underpowered for the observed effect size. This increases the risk of Type II errors. Consequently, non-significant findings should be interpreted with caution. More importantly, while the discussion proposed several mechanisms for the observed U-shaped performance curve, key variables related to these mechanisms were not directly measured. The study lacked systematic assessment of GI symptoms, direct measurement of substrate oxidation rates, and monitoring of hydration status. Consequently, the proposed explanations regarding GI distress and metabolic flexibility, while plausible, remain speculative without direct empirical evidence from this cohort. While the between-subjects design minimized the risk of participants comparing doses, potential variations in beverage sweetness and viscosity between the highest and lowest concentrations may have partially compromised the blind. The psychological impact of this, if any, on performance is unknown. Additionally, the modified 2 × 30 min exercise protocol, though necessary for feasibility, may elicit different physiological and performance responses compared to a continuous 1-h effort. Finally, the simultaneous testing of multiple participants is a limitation. On some occasions, up to three individuals performed the protocol concurrently. Auditory and visual cues of effort from co-participants may have introduced a competitive or pacing element (51, 53). This factor could have influenced individual work output, despite efforts to blind performance feedback.

Future research should aim to address these limitations to build a more mechanistic understanding. It is recommended that subsequent studies incorporate validated GI symptom questionnaires to quantify perceptual GI stress (54, 55). To directly test the metabolic flexibility hypothesis, substrate utilization should be measured using indirect calorimetry (56, 57). For even greater precision in determining the oxidation rates of exogenous and endogenous fuels, the use of stable isotope tracers is warranted (58–60). Replicating this detailed dose–response protocol in elite and female athletes is also a crucial next step. Finally, exploring whether this non-linear performance curve exists with different CHO-to-PRO ratios could further refine nutritional recommendations for endurance athletes.

From a practical standpoint, these findings provide critical guidance for athletes and coaches. The results challenge the conventional ‘more is better’ approach to in-exercise supplementation. For high-intensity rowing of approximately 1-h duration, a lower-dose strategy (e.g., ~0.5 g/kg/h CHO) appears more effective than consuming nutrients at rates approaching established ceilings. Athletes should avoid assuming that maximal recommended doses will yield maximal performance. Instead, an individualized approach is essential. Nutritional strategies should be systematically trialed and titrated based on objective performance outcomes, rather than on transient physiological sensations or generic guidelines. An optimal strategy balances energy provision with individual physiological tolerance to maximize performance.

## 5 Conclusion

This study investigated the dose–response effects of a 4:1 CHO–PRO on prolonged rowing performance. A randomized, double-blind, parallel-group trial was conducted involving 171 male university students. During a standardized 1-h rowing protocol, participants ingested one of eight distinct supplement doses, with CHO content ranging from 0.5 to 1.2 g/kg/h. Performance was quantified by the total distance rowed during the exercise test.

A non-linear, U-shaped dose–response relationship was observed, with the lowest supplement dose (0.5 g/kg/h CHO) yielding significantly better performance than several higher doses. This finding suggests that for this type of exercise, performance decrements at higher intake levels may be due to increased GI load and impaired metabolic efficiency. The momentary physiological benefits seen with higher doses did not translate to improved overall performance. This indicates that performance is an integrated outcome not dictated by isolated physiological markers. Practically, these findings challenge the ‘more is better’ paradigm for in-exercise nutrition. For prolonged, high-intensity rowing, a lower dose of CHO–PRO proved superior to higher doses.

## Data Availability

The raw data supporting the conclusions of this article will be made available by the authors, without undue reservation.
